# Purified Cornel Iridoid Glycosides Attenuated Oxidative Stress Induced by Cerebral Ischemia-Reperfusion Injury via Morroniside and Loganin Targeting Nrf2/NQO-1/HO-1 Signaling Pathway

**DOI:** 10.3390/cells14151205

**Published:** 2025-08-06

**Authors:** Zhaoyang Wang, Fangli Xue, Enjie Hu, Yourui Wang, Huiliang Li, Boling Qiao

**Affiliations:** 1 Key Laboratory of Resource Biology and Modern Biotechnology in Western China, Ministry of Education, Northwest University, No. 229 Taibai North Road, Xi’an 710069, China; wzy17864190778@163.com (Z.W.);; 2 Shaanxi Traditional Chinese Medicine Innovation Engineering Technology Research Center, No. 229 Taibai North Road, Xi’an 710069, China; 3 Wolfson Institute for Biomedical Research, Division of Medicine, Faculty of Medical Sciences, University College London, London WC1E 6BT, UK; huiliang.li@ucl.ac.uk

**Keywords:** cerebral ischemia-reperfusion injury, oxidative stress, morroniside, loganin, purified cornel iridoid glycosides, Nrf2 signaling pathway

## Abstract

Oxidative stress significantly contributes to the exacerbation of brain damage during cerebral ischemia-reperfusion injury (CIR/I). In our previous study, purified cornel iridoid glycoside (PCIG), consisting of morroniside (MOR) and loganin (LOG), showed neuroprotective effects against CIR/I. To further explore the antioxidative effects and underlying molecular mechanisms, we applied PCIG, MOR, and LOG to rats injured by middle cerebral artery occlusion/reperfusion (MCAO/R) as well as H_2_O_2_-stimulated PC12 cells. Additionally, the molecular docking analysis was performed to assess the interaction between the PCIG constituents and Kelch-like ECH-associated protein 1 (Keap1). The results showed that the treated rats experienced fewer neurological deficits, reduced lesion volumes, and lower cell death accompanied by decreased levels of malondialdehyde (MDA) and protein carbonyl, as well as increased activities of superoxide dismutase (SOD) and glutathione peroxidase (GSH-Px). In H_2_O_2_-stimulated PC12 cells, the treatments decreased reactive oxygen species (ROS) production, mitigated mitochondrial dysfunction, and inhibited mitochondrial-dependent apoptosis. Moreover, the treatments facilitated Nuclear factor (erythroid-derived 2)-like 2 (Nrf2) translocation into the nucleus and selectively increased the expression of NAD(P)H quinone oxidoreductase 1 (NQO-1) and heme oxygenase 1 (HO-1) through MOR and LOG, respectively. Both MOR and LOG demonstrated strong binding affinity to Keap1. These findings suggested that PCIG, rather than any individual components, might serve as a valuable treatment for ischemic stroke by activating the Nrf2/NQO-1 and Nrf2/HO-1 signaling pathway.

## 1. Introduction

Ischemic stroke accounts for about 80% of all stroke cases and has high mortality, disability, and recurrence rates [1]. Early recovery of regional cerebral blood flow is crucial for treating ischemic stroke, as it enables timely recanalization of blood vessels and improves collateral circulation during the acute phase [2,3]. Clinical studies have illustrated that thrombolytic treatment can effectively restore cerebral ischemia, preserve the ischemic penumbra, and significantly improve brain function and prognosis. However, the restoration of blood flow can also trigger a complex chain of molecular events that further worsen brain damage and neurological impairment, leading to a condition known as cerebral ischemia reperfusion/injury (CIR/I) [4].

The development of CIR/I is a complex process involving various factors such as the accumulation of free radicals, cell apoptosis, immune dysfunction, excessive release of excitatory amino acid toxicity, intracellular calcium overload, and so on [5]. Notably, oxidative stress plays a significant role in causing neuro-cell death in CIR/I [6]. Oxidative stress refers to an imbalance between the generation of reactive oxygen species (ROS) and the ability of cells to detoxify them, which could be caused by either increased ROS levels or decreased activities of antioxidants. Under normal physiological conditions, ROS are primarily produced in the mitochondria and maintained within well-defined limited levels for cell growth and metabolism in the brain. However, when blood and oxygen flow are restored to ischemic and hypoxic brain tissue, oxidative stress is activated, promoting more enzyme-catalyzed oxidation reactions. These can result in the overproduction of excessive ROS, disrupting the balance between free radical production and scavenging [7]. The excessive ROS can further accelerate lipid accumulation, cause DNA and protein denaturation, and subsequently induce mitochondrial dysfunction, ultimately causing brain cell death [8]. Enzymatic antioxidants, superoxide dismutase (SOD), catalase (CAT), and glutathione peroxidase (GSH-Px) are the representative primary enzymes and play an important role in scavenging excessive ROS. SOD can remove ROS by catalyzing the disproportionation of superoxide anion radicals to O_2_ and H_2_O_2_, with CAT further converting H_2_O_2_ into H_2_O and O_2_ [9]. GSH-Px can protect the integrity of cell membrane structure and function by using glutathione (GSH) as a reducing agent in catalyzing the reduction of H_2_O_2_ to H_2_O [10]. Therefore, antioxidants capable of scavenging excessive ROS are considered valuable targets for CIR/I treatment.

Nuclear factor (erythroid-derived 2)-like 2 (Nrf2) is a central regulator of antioxidant systems that directly antagonizes free radical effects and regulates cellular antioxidant activity by controlling the transcription of many enzymatic antioxidants. In homeostatic conditions, Nrf2 exists as components of inactive cytoplasmic complexes bound by Kelch-like ECH-associated protein 1 (Keap1). Upon activation, Nrf2 is released and transported into the nucleus, where it binds to the antioxidant response element (ARE) in the promoter region of target genes. This binding triggers the transcriptional activation of various antioxidants, like NAD(P)H quinone dehydrogenase (NQO-1) or heme oxygenase 1 (HO-1), that could enhance cellular resistance to oxidative stress. Studies have shown that Nrf2 knockout mice exhibited reduced expression of antioxidants and diminished neuroprotective effects in stroke animal models [11]. Therefore, activating the Nrf2 signaling pathway has been proposed as a primary defense mechanism against oxidative stress during conditions of CIR/I [12]. Drugs that effectively activate Nrf2 and stimulate the transcription of downstream antioxidant enzyme genes have been considered promising candidates for neuroprotection in CIR/I outcomes [13,14].

**Figure 1:**
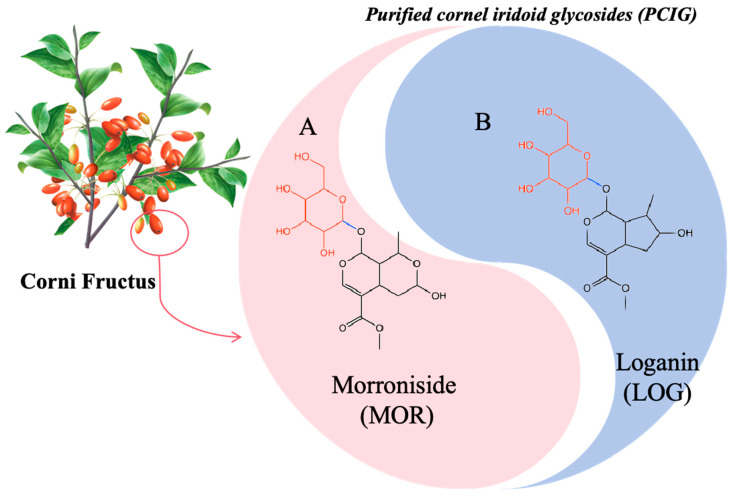
The composition of purified cornel iridoid glycosides.

Corni Fructus (*Cornus officinalis* Sieb. et Zucc.) is a traditional Chinese medicine with functions that nourish the liver and kidneys. Our previous study demonstrated that ethanol extracts of Corni Fructus provided protection against inflammation in CIR/I rats [15]. In Corni Fructus, cornel iridoid glycosides are the main components, in which morroniside (MOR) and loganin (LOG) (Figure 1) are often used as chemical markers for quality control [16]. Studies have shown that these components have protective effects against liver injury in mouse models of non-alcoholic fatty liver disease and against kidney damage in diabetic mice [17,18]. These protective effects were attributed to their antioxidative stress activities, such as reducing the levels of ROS-related toxic products and increasing the levels of antioxidant enzymes [19]. In our previous study, the purified cornel iridoid glycosides (PCIG), composed of MOR and LOG, showed beneficial effects in reducing brain edema in CIR/I rats [20]. We presumed that the neuroprotective effects might stem from its constituents’ activities in combating oxidative stress.

In this study, PCIG, MOR, and LOG were investigated for the effectiveness of reducing oxidative stress in rats injured by middle cerebral artery occlusion/reperfusion (MCAO/R). Additionally, molecular docking analysis and H_2_O_2_-stimulated PC12 cells were utilized to explore the molecular mechanisms underlying the impact of PCIG, MOR, and LOG on ROS-mediated apoptosis and the activation of the Nrf2 signaling pathway.

## 2. Materials and Methods

### 2.1. Drugs

Purified cornel iridoid glycosides (PCIG) were extracted from the dried sarcocarp of *Cornus officinalis* Sieb.et Zucc (Shaanxi Pharmaceutical Holding Group Co., Ltd., Xi’an, China), which, in accordance with the provisions of the Chinese Pharmacopoeia (2020 edition), and purified by using the method as described in our previous study [20]. The content of MOR and LOG in the PCIG was determined by reverse-phase high-performance liquid chromatography (HPLC).

Morroniside (HM095359, purity ≥ 98%) and loganin (HL163398, purity ≥ 98%) were provided by Baoji Herbest Biotechnology Co., Ltd. (Baoji, China).

### 2.2. Animal, Middle Cerebral Artery Occlusion Injury (MCAO/R) and Drug Treatment

Male Sprague Dawley (SD) rats were purchased from Chengdu Dashuo Experimental Animal Co., Ltd. (Chengdu, China) (certificate No.SCXK-2020-030) and housed in a controlled environment with a constant temperature of 22 ± 2 °C, humidity of 60 ± 5%, and a 12/12 h light/dark cycle. The rats were given free access to standard laboratory chow and water during the study. All procedures for the care and handling of animals used in the study were approved by the Ethics Committee of Northwest University for Animal Experimentation (ACCNU-2020-0016) and carried out according to the Guideline for Animal Experimentation of Northwest University and the use of Laboratory animals (NIH Publications No. 8023, revised 1978).

After one-week adaptive feeding, rats (230–250 g body weight) were randomly divided into seven groups: SHAM Group, MCAO/R Group, low-dose of the PCIG (L-PCIG, 15 mg/kg, i.g.), medium-dose of the PCIG (M-PCIG, 30 mg/kg, i.g.), high-dose of the PCIG (H-PCIG, 60 mg/kg, i.g.) groups, morroniside group (MOR, 46.2 mg/kg, i.v.) and loganin group (LOG, 18.5 mg/kg, i.v.) with eight rats in each group. PCIG was administered in distilled water by the intragastric route once daily for 5 days. Morroniside and loganin were dissolved in normal saline and injected via the tail vein two hours before the modeling procedure. The MCAO/R and SHAM rats received an equal volume of distilled water. On day 5, two hours after the last administration of the drugs, the MCAO/R procedure was performed on the rats, with reperfusion initiated 1 h after the MCAO as described in our previous study [20]. After 24 h, the rats were evaluated for neurological function, followed by euthanization to collect brains for further experiments. The specific sample numbers for each experiment were indicated in the figure legend.

### 2.3. Neurological Deficit

Neurological function was scored using the modified Zea Longa method. Briefly, the neurological function score was graded on a scale of 0 to 4: 0 = no deficits; 1 = failure to extend forepaw fully; 2 = turning or shifting to the paralyzed side when walking; 3 = no spontaneous motor activity or collapsed to the paralyzed side when walking; 4 = die or unable to move autonomously.

### 2.4. 2,3,5-Triphenyltetrazolium Hydrochloride (TTC) Staining

Briefly, the rat brains were cut into five slices, followed by staining in 4% TTC solution (G1017) in the dark at 37 °C for 30 min. The stained brain slices were fixed with 4% paraformaldehyde solution (G1101) for 24 h, and photographed for later analysis by using Image J analysis systems (National Institutes of Health, USA). Chemical reagents were purchased from Wuhan Servicebio Biotechnology Co., Ltd. (Wuhan, China). The infarct volume ratio was calculated using the following formula.

Infarction rate = infarction area/whole brain area × 100%.

### 2.5. Brain Water Content Measurement

The rat brains recorded with the wet weight were dried in a 110 °C oven for 24 h, followed by measuring with the dry weight. Water content in the brain was calculated using the following formula.

Brain water content = (wet weight − dry weight)/wet weight × 100%.

### 2.6. H&E and TUNEL Staining

The rat brains fixed in polyformaldehyde (G1101, Wuhan Servicebio Biotechnology Co., Ltd., Wuhan, China) were paraffin-embedded and cut at 4 μm for subsequent staining. The section stained was photographed under a fluorescence microscope (Olympus, Tokyo, Japan).

For hematoxylin and eosin (H&E) staining, the section was stained with the method described in our previous study [21].

For terminal deoxynucleotidyl transferase dUTP nick end labeling (TUNEL) staining, the section was dewaxed and dehydrated, then stained using fluorescein (FITC) TUNEL apoptosis detection kit (G1501, Wuhan Servicebio Biotechnology Co., Ltd., Wuhan, China) according to the manufacturer’s instructions. DAPI solution (P1301, Shanghai Beyotime Biotechnology Co., Ltd., Shanghai, China) was used in the last step.

### 2.7. Cell Culture and Treatment

PC12 cells (American Type Culture Collection, Manassas, VA, USA) were cultured in Dulbecco’s modified eagle medium (DMEM) high glucose culture medium (Gbico, Thermo Fisher Scientific, MA, USA) containing 10% fetal bovine serum (Lanzhou Roya Bio-technology Co., Ltd., Lanzhou, China) at 37 °C in 5% carbon dioxide in a humidified incubator. Exponential growth of cells was used for all the experiments.

For the treatment, exponentially growing PC12 cells were seeded in culture plates for 24 h, then treated with or without H_2_O_2_ (3587191, Sigma-Aldrich, STL, USA) or drugs for a further 24 h. Drug stock solution was added directly to the cell culture media to attain corresponding concentrations.

### 2.8. MTT Assays

The cells were cultured in medium containing 5 mg/mL of MTT (A600799, Sangon Biotech (Shangai) Co., Ltd.) for 4 h at 37 °C and 5% carbon dioxide incubator, The cells in dimethyl sulfoxide (DMSO, A600163, Sangon Biotech (Shangai) Co., Ltd.) were detected with optical density at 490 nm by enzyme labeling instrument (Nanjing Detie Experimental equipment Co., Ltd., Nanjing, China). Cell viability was calculated as percentage value normalized to the untreated PC12 cells.

### 2.9. Determination of ROS Levels

PC12 cells were stained using DCFH-DA (S0033S, Beyotime Biotechnology Co., Ltd., Shanghai, China) for 20 min in the dark, and imaged under a laser confocal microscope (Leica, Wetzlar, Germany).

### 2.10. Determination of Mitochondrial Function

PC12 cells were stained using mitochondrial membrane potential (JC-1) detection kit (M8650, Beijing Solarbio Science & Technology Co., Ltd., Beijing, China) at 37 °C for 30 min according to the manufacturer’s instructions. The stained cells were photographed under a fluorescence microscope (Olympus, Tokyo, Japan).

### 2.11. Immunofluorescence Detection

PC12 cells were fixed with paraformaldehyde, treated with 0.15% Triton X-100 (T8200, Beijing Solarbio Science & Technology Co., Ltd., Beijing, China) at 37 °C for 10 min and then incubated overnight at 4 °C with Nrf2 primary antibody (A0674, 1:200, ABclonal Biotechnology Co., Ltd., Wuhan, China). After washing off the primary antibody with phosphate-buffered saline (PBS), cells were incubated in the dark for 1 h with the Cy3-conjugated goat anti-rabbit IgG (AS007, 1:500, ABclonal Biotechnology Co., Ltd., Wuhan, China). The secondary antibody was washed off with PBS, counterstained with DAPI, and observed under a laser confocal microscope (Leica, Wetzlar, Germany).

### 2.12. Western Blotting

Cells were extracted with protein using lysis buffer (V900854, Sigma-Aldrich, STL, USA). The protein concentration was determined using Bradford agent (C503031, Sangon Biotech (Shanghai) Co., Ltd.). The proteins were resolved on a 10–12% sodium dodecyl sulfate-polyacrylamide gel electrophoresis, followed by transferring to polyvinylidene fluoride membrane (Merck Millipore, IPVH00010). The membrane was blocked with 5% skimmed milk powder (A600669, Sangon Biotech (Shanghai) Co., Ltd.) at room temperature for 1 h, and then incubated overnight at 4 °C with the following primary antibodies (ABclonal Biotechnology Co., Ltd., Wuhan, China): BCL-2, (A19693, 1:500), BAX (A19684, 1:500), Caspase-3 (A11319, 1:500), HO-1 (A19062 1:500), NQO-1 (A19586, 1:500), mouse anti-β-actin (A5316, 1:2000, Sigma-Aldrich, STL, USA). Then, the membrane was incubated with the second antibody at room temperature for 1 h, and visualized by enhanced fluorescence substrate (P0018AS, Beyotime Biotechnology Co., Ltd., Shanghai, China). The protein expression was quantified based on the band density analyzed using ImageJ software Version 1.54.

### 2.13. Biochemical Analysis

Rat brain tissues or PC12 cells were extracted with the proteins, and the levels of SOD (A001-3-2), GSH-Px (A005-1-2), GSH (A006-2-1), protein carbonyl (A087-1-2) and MDA (A003-1-2) were detected by using commercially available kits, following the manufacturer’s protocol (Nanjing Jiancheng Institute of Biological Engineering, Nanjing, China).

### 2.14. Molecular Docking

The structures of morroniside and loganin 3D were obtained through Pubchem. In the RCSB PDB database (https://www.Rcsb.Org/ (accessed on 9 May 2025)), to obtain the crystal 3D structure of the target protein (PDB code: 4L7B), using AutoDockTools 1.5.6 software removes water molecules and excess small molecular ligands from the core targets and hydrogenates them. 3D structures of the compounds from the PUBCHEM database were downloaded. AutoDockTools 1.5.6 software was used for molecular docking, and Discovery Studio 2019 Client software was used for visual analysis.

### 2.15. Statistical Analysis

Statistical analysis and mapping were performed using GraphPad Prism 9.0 (GraphPad Software, La Jolla, CA, USA). The normality of the dependent variable within groups was assessed using the Shapiro-Wilk test, and the homogeneity of variances between groups was evaluated using Brown–Forsythe test. Single-factor analysis of variance (ANOVA) was used to compare the differences between multiple samples. And the *p* values were subjected further to Bonferroni’s correction. All data were expressed as means ± SD. *p* < 0.05 was used to determine the significance of differences. All *p* values shown in the study are referred to the post-correction values.

## 3. Results

### 3.1. Protected the Rats Against MCAO/R-Induced Brain Damages

**Figure 2:**
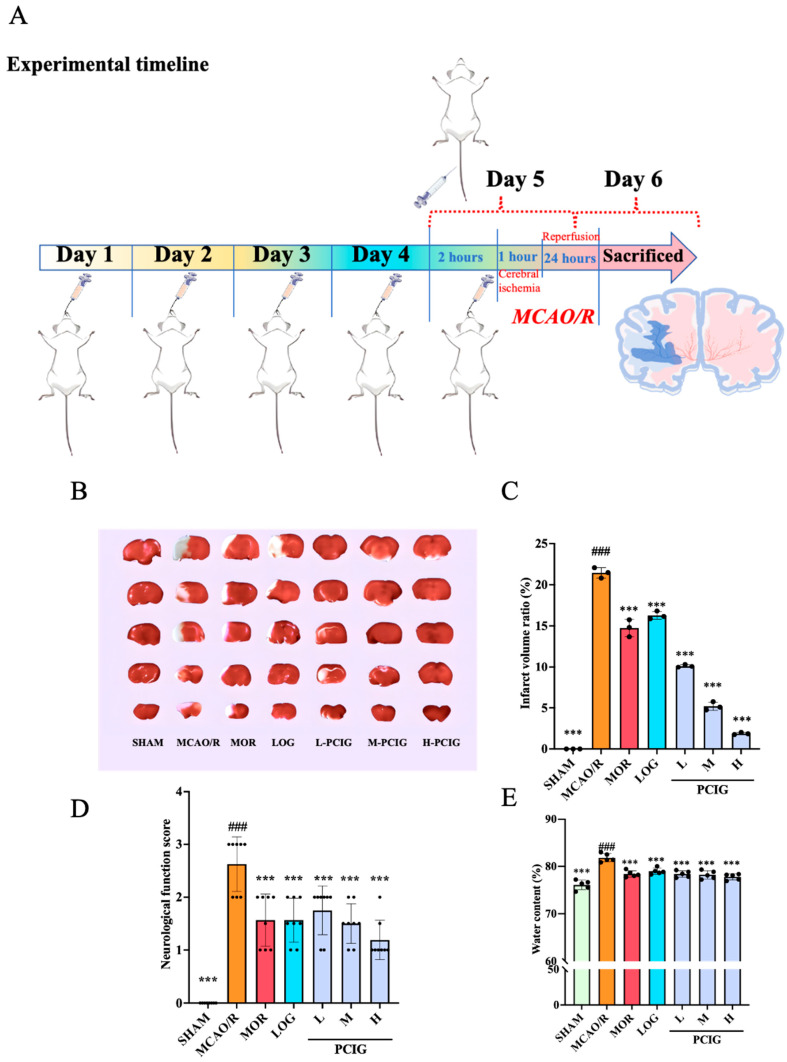
Protective effect of MOR, LOG and PCIG on cerebral ischemia-reperfusion injury in rats. (**A**) Timeline flowchart for the animal experiments. (**B**) Representative brain sections with TTC staining. (*n* = 3). (**C**) Infarct volume ratio based on TTC staining. (*n* = 3). (**D**) Neurological scores. (*n* = 8). (**E**) Brain water content of the rats. (*n* = 5). The columns and error bars were represented as means ± SD. ### *p* < 0.001 vs. the SHAM group, *** *p* < 0.001 vs. the MCAO/R group.

Oral administration is a widely used method for PCIG delivery. However, given the reported lower bioavailability associated with this route, we employed intravenous administration of MOR and LOG for comparison. The experimental timeline and treatment methods are outlined in Figure 2A.

Cerebral infarction size is often used as the primary endpoint to evaluate the degree of brain injury. As shown in Figure 2B,C, the SHAM rats exhibited no infarcts, as indicated by all the sections staining red in the TTC solution. This infarct volume ratio was defined as the minimum ratio at 0%. However, rats in the MCAO/R group showed significant cerebral infarction, which appeared as white areas in the sections of brain tissue. Compared with the SHAM group, the MCAO/R group displayed a considerable increase in brain infarction, with an infarct volume ratio of 21.6% (*p* ≤ 0.0001). Compared with the MCAO/R rats, the rats in MOR, LOG, L-PCIG, M-PCIG, and H-PCIG groups showed a significant reductions in the infarction size with a decrease of 32.6% (*p* ≤ 0.0001), 24.5% (*p* ≤ 0.0001), 53.0% (*p* ≤ 0.0001), 76.4% (*p* ≤ 0.0001) and 91.2% (*p* ≤ 0.0001), respectively.

The neurobehavioral score of rats can directly reflect the degree of motor dysfunction. In the SHAM group, the rats exhibited no deficits, achieving a score of 0 (Figure 2D). Compared with the SHAM group, rats in the MCAO/R group displayed notable symptoms, such as turning toward the paralyzed side or even falling to the opposite side of the lesion while walking. This resulted in a neurobehavioral score of 2.5 (*p* ≤ 0.0001), indicating severe neurological deficits. In comparison with the MCAO/R group, the treatments of MOR, LOG, L-PCIG, M-PCIG, and H-PCIG group led to significant improvements in neurological functions with decreases of 42.3% (*p* = 0.0018), 38.5% (*p* = 0.005), 30.8% (*p* = 0.0343), 42.3% (*p* = 0.0018), and 53.8% (*p* ≤ 0.0001), respectively.

The brain water content can serve as an indicator of the presence of brain edema (Figure 2E). Compared to the SHAM group, the brain water content in MCAO/R rats increased by 7.6% (*p* ≤ 0.0001). Compared to MCAO/R rats, the brain water content in MOR, LOG, L-PCIG, M-PCIG, and H-PCIG groups decreased by 4.2% (*p* ≤ 0.0001), 3.5% (*p* ≤ 0.0001), 4.2% (*p* ≤ 0.0001), 4.4%(*p* ≤ 0.0001) and 5.0% (*p* ≤ 0.0001), respectively.

### 3.2. Improvement on Histopathological Changes in MCAO/R Rats

**Figure 3:**
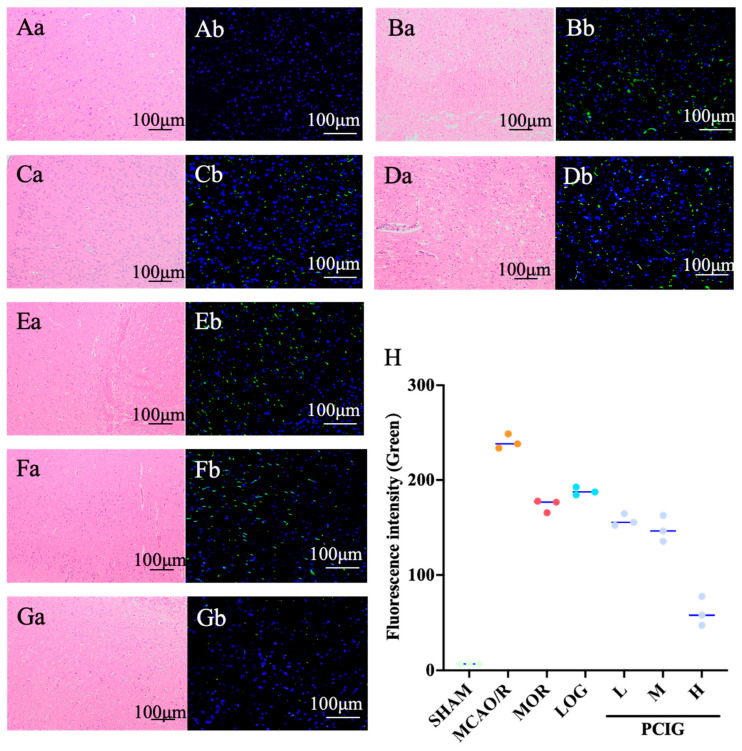
Histopathological analysis on brain tissues of the rats involved in the SHAM (**A**), MCAO/R (**B**), MOR (**C**), LOG (**D**), L-PCIG (**E**), M-PCIG (**F**) and H-PCIG (**G**) groups. Representative (**a**) H&E staining. (**b**) TUNEL staining. (**H**) Number of green fluorescence cells based on TUNEL staining. (*n* = 3).

To examine the histopathological changes in the rat brains, H&E staining was performed on the brain tissues (Figure 3). In the SHAM group (Figure 3Aa), the rats exhibited compact and neatly arranged neurons with intact nuclei and cell structures. In the MCAO/R group (Figure 3Ba), the cell bodies were swollen and deformed, and the nucleolus appeared withered or missing. The interstitial spaces between cells were loose, and the tissue showed significant edema and vacuolation. Following treatment with MOR (Figure 3Ca), LOG (Figure 3Da), L-PCIG (Figure 3Ea), M-PCIG (Figure 3Fa), and H-PCIG (Figure 3Ga), the rats showed marked improvement in histopathological changes in the brain. The arrangement of neurons in the cerebral cortex returned to normal, with a noticeable reduction in vacuolated cells and nuclear pyknosis. Furthermore, the effectiveness of the PCIG treatments increased with the higher dosages of PCIG used.

To observe cellular damage in the brain, tunnel staining was performed on sections of rat brain tissue. Dead cells, identified by DNA strand breaks, were labeled with green fluorescence. The SHAM rats (Figure 3Ab) showed no signs of dead cells in the brain tissue. In contrast, a significant number of apoptotic cells were observed in the cortical area of the MCAO/R rat brains (Figure 3Bb). In the rat brain tissues collected from MOR (Figure 3Cb), LOG (Figure 3Db), L-PCIG (Figure 3Eb), M-PCIG (Figure 3Fb), and H-PCIG (Figure 3Gb) groups, the number of apoptotic cells was significantly less. PCIG treatments showed prominent effects in decreasing the number of apoptotic cells in a dose-dependent manner.

### 3.3. Enhanced the Antioxidant Capacity of MCAO/R Rats

**Figure 4:**
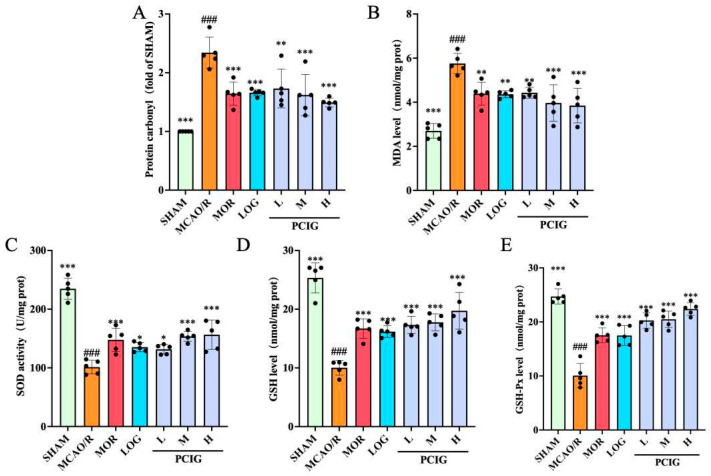
Biochemical analysis on rat brains collected from SHAM, MCAO/R, MOR, LOG, L-PCIG, M-PCIG and H-PCIG groups. Levels of (**A**) protein carbonyl; (**B**) MDA; (**C**) SOD activity; (**D**) GSH; (**E**) GSH-Px. The columns and error bars were represented as means ± SD. ### *p* < 0.001 vs. the SHAM group, * *p* < 0.05 vs. the MCAO/R group, ** *p* < 0.01 vs. the MCAO/R group, *** *p* < 0.001 vs. the MCAO/R group (*n* = 5).

MDA and protein carbonyl are oxidative products related to ROS and are classified as biologically toxic substances [22]. MDA is a by-product of lipid peroxidation, while protein carbonyl results from irreversible non-enzymatic oxidation or carbonylation of proteins. The accumulation of these products could lead to neuronal cell dysfunction and cell death. To assess the effect of the samples on antioxidant activity, the levels of these oxidative products were measured. As shown in Figure 4A,B, MCAO/R rats had increases of 1.34-fold in protein carbonyl levels (*p* ≤ 0.0001) and 1.13-fold in MDA levels (*p* ≤ 0.0001) compared to SHAM rats. Treatment with MOR, LOG, and PCIG resulted in reductions in these elevated levels. Compared with the MCAO/R rats, the rats in MOR, LOG, L-PCIG, M-PCIG, and H-PCIG groups showed decreased levels of protein carbonyl by 29.8% (*p* = 0.0002), 29.1% (*p* = 0.0003), 26.1% (*p* = 0.0011), 30.8% (*p* = 0.0001) and 36.2% (*p* ≤ 0.0001), respectively. For MDA levels, there was a decrease of 23.7% (*p* = 0.0021), 24.2% (*p* = 0.0017), 23.5% (*p* = 0.0029), 31.1% (*p* ≤ 0.0001), and 33.2% (*p* ≤ 0.0001) in MOR, LOG, L-PCIG, M-PCIG and H-PCIG groups, respectively. These results indicate that PCIG treatments lead to a significant dose-dependent decrease in the levels of MDA and protein carbonyl.

Additionally, the antioxidant defense system comprises multiple antioxidant enzymes that form the first line of defense against oxidative stress, serving as a crucial protective barrier. This line of defense plays an irreplaceable role in the dismutation of excessive ROS [23]. Representative indicators of this system include SOD, GSH-Px, and GSH. Thus, changes in the levels of these indicators in the rat brains were investigated in this study (Figure 4C–E). Compared to the SHAM rats, the MCAO/R rats showed significant decreases of 56.8% (*p* ≤ 0.0001), 60.4% (*p* ≤ 0.0001), and 59.7% (*p* ≤ 0.0001) in the levels of SOD, GSH, and GSH-Px, respectively. Compared with the MCAO/R group, the rats in the MOR, LOG, L-PCIG, M-PCIG, and H-PCIG groups showed an increased SOD clearance rate of 45.8% (*p* = 0.0004), 33.5% (*p* = 0.0109), 29.9% (*p* = 0.0274), 51.5% (*p* ≤ 0.0001), 54.4% (*p* ≤ 0.0001), respectively. The levels of GSH increased by 66.7% (*p* ≤ 0.0001), 61.3% (*p* ≤ 0.0001), 72.6% (*p* ≤ 0.0001), 77.1% (*p* ≤ 0.0001), and 96.6% (*p* ≤ 0.0001) in MOR, LOG, L-PCIG, M-PCIG and H-PCIG rats, respectively. As for the levels of GSH-Px, there was an increase of 1.74-fold (*p* ≤ 0.0001), 1.73-fold (*p* ≤ 0.0001), 2.01-fold (*p* ≤ 0.0001), 2.03-fold (*p* ≤ 0.0001), and 2.22-fold (*p* ≤ 0.0001) in MOR, LOG, L-PCIG, M-PCIG, and H-PCIG rats, respectively. Notably, the PCIG treatments showed more pronounced effects, enhancing the levels in a dose-dependent manner.

### 3.4. Protected PC12 Cells Against H_2_O_2_-Induced Cell Apoptosis

To effectively mitigate the impact of PCIG metabolites generated during oral administration, we conducted a cell-based experimental study aimed at exploring the effects and underlying mechanisms of the individual constituents of PCIG.

PC12 cells are derived from transplantable rat adrenal pheochromocytoma and exhibit the physiological and biochemical characteristics of sympathetic neurons. Hydrogen peroxide is commonly used to induce oxidative injury in cells [24].

**Figure 5:**
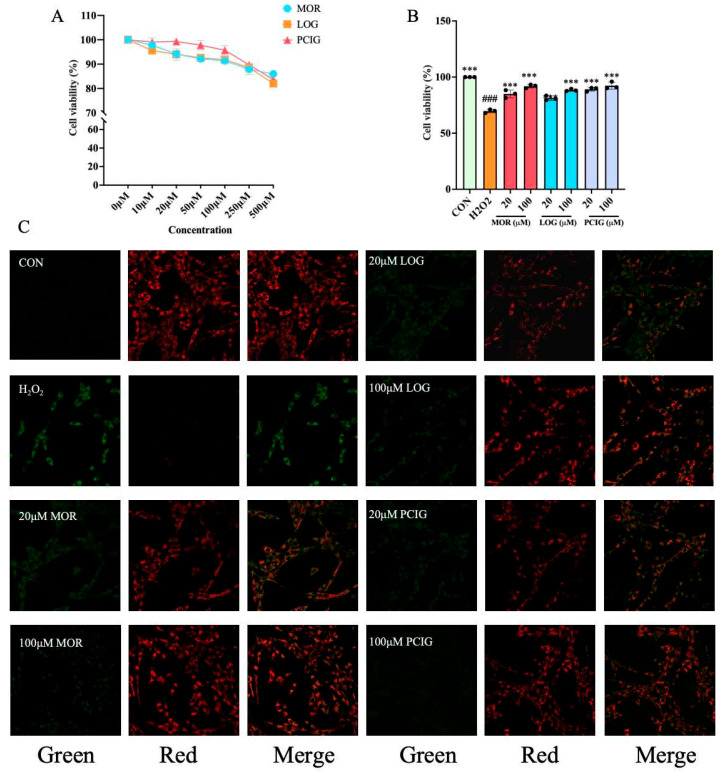
Protective effects of MOR, LOG and PCIG on PC12 cells against H_2_O_2_-induced cell apoptosis. Viability of (**A**) PC12 cells treated with the samples. (**B**) H_2_O_2_-stimulated PC12 cells were treated with the samples. (**C**) mitochondrial membrane potential of the cells. The columns and error bars were represented as means ± SD. ### *p* < 0.001 vs. the CON, *** *p* < 0.001 vs. the H_2_O_2_ (*n* = 3).

An MTT assay was conducted to evaluate the viability of PC12 cells treated with PCIG, MOR, and LOG at varying doses, ranging from 10 µM to 500 µM. Figure 5A illustrates the different doses of MOR, LOG, and PCIG given to achieve final concentrations of 10, 20, 50, 100, 250, and 500 μM. When the concentration of MOR, LOG, and PCIG was less than 100 µM, 100 µM, and 250 µM, respectively, the survival rate of PC12 cells was over 90%. This indicates that there were no significant toxic effects on PC12 cells within this concentration range. Therefore, in further experiments, H_2_O_2_-stimulated PC12 cells were treated with two doses (20 μM and 100 μM) of PCIG, MOR, and LOG.

Stimulation with H_2_O_2_ at a concentration of 150 μM led to a reduction in cell viability, decreasing it to 70% (Figure 5B). Treatment with PCIG, MOR, or LOG significantly increased cell viability to over 80% in a dose-dependent manner. Notably, PCIG treatments achieved the highest level of cell viability at over 89.16%, demonstrating the most prominent effect.

Mitochondria are the main organelles responsible for energy metabolism in cells and play a key role in mediating cell apoptosis. Loss of mitochondrial transmembrane potential (ΔΨ_M_) could result in mitochondrial dysfunction, triggering intrinsic apoptosis by modulating a series of apoptotic proteins, including BAX, BCL-2, and Caspase-3 [25].

JC-1 is a membrane-permeant dye that selectively accumulates in the mitochondria. It changes its emission color from green to red as the ΔΨ_M_ becomes more polarized. Untreated PC12 cells showed a polarized ΔΨ_M_ and exhibited strong red fluorescence. H_2_O_2_-stimulated cells exhibited strong green fluorescence, suggesting a loss of ΔΨ_M_. Treatment with PCIG, MOR, or LOG at two concentrations caused the H_2_O_2_-stimulated cells to shift from green to red fluorescence (Figure 5C).

**Figure 6:**
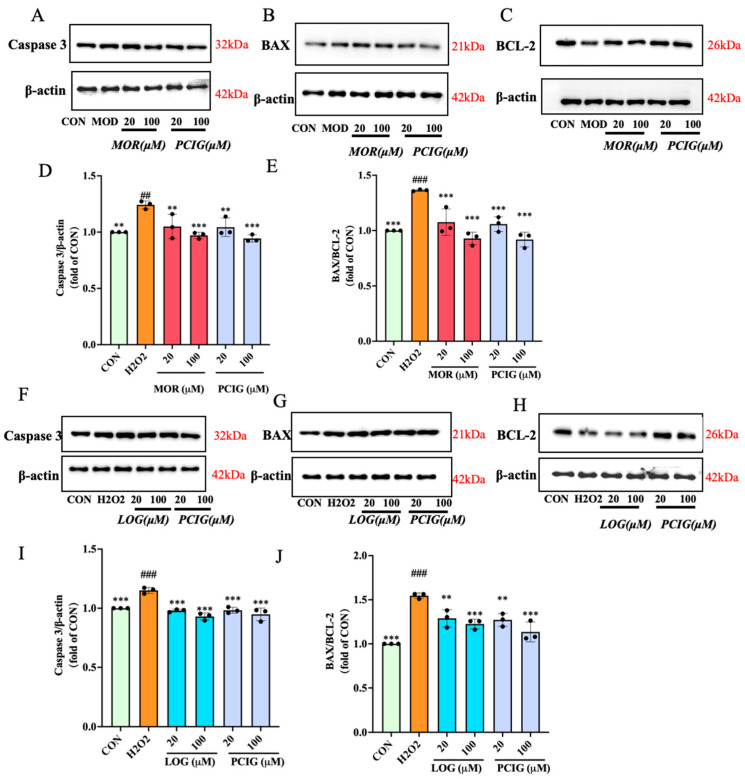
Protective effects of MOR, LOG and PCIG on PC12 cells against H_2_O_2_-induced cell apoptosis. The protein levels of (**A**,**D**) Caspase-3, (**B**,**C**,**E**) BAX, BCL-2 and BAX/BCL-2 in PC12 cells treated with MOR and PCIG. The protein levels of (**F**,**I**) Caspase-3, (**G**,**H**,**J**) BAX, BCL-2 and BAX/BCL-2 in PC12 cells treated with LOG and PCIG. The columns and error bars were represented as means ± SD. ## *p* < 0.01 vs. the CON, ### *p* < 0.001 vs. the CON, ** *p* < 0.01 vs. the H_2_O_2_, *** *p* < 0.001 vs. the H_2_O_2_ (*n* = 3).

To investigate the apoptosis induced, we examined the expression levels of apoptosis-related proteins, including pro-apoptotic proteins BAX and Caspase-3, as well as anti-apoptotic protein BCL-2. Compared to the untreated PC12 cells (control group, CON), H_2_O_2_ stimulation slightly increased the expression of Caspase-3. We presumed that H_2_O_2_ stimulation might induce initial mitochondrial stress (as shown in Figure 6), leading to transcriptional upregulation of these factors as a feedforward loop. Furthermore, the decreased expression of BCL-2 could be observed, resulting in an increase in the ratio of BAX/BCL-2 by 1.36-fold. Compared to the H_2_O_2_-stimulated cells, treatments with MOR, LOG or PCIG at two concentrations resulted in a decrease in the expression of Caspase-3. For the BAX/BCL-2 ratio, the decrease in MOR, LOG or PCIG-treated cells was 16.2–19.7%, 16.6–20.7% or 16.5–26.6%, respectively.

### 3.5. Enhanced Activities on Scavenging ROS

**Figure 7:**
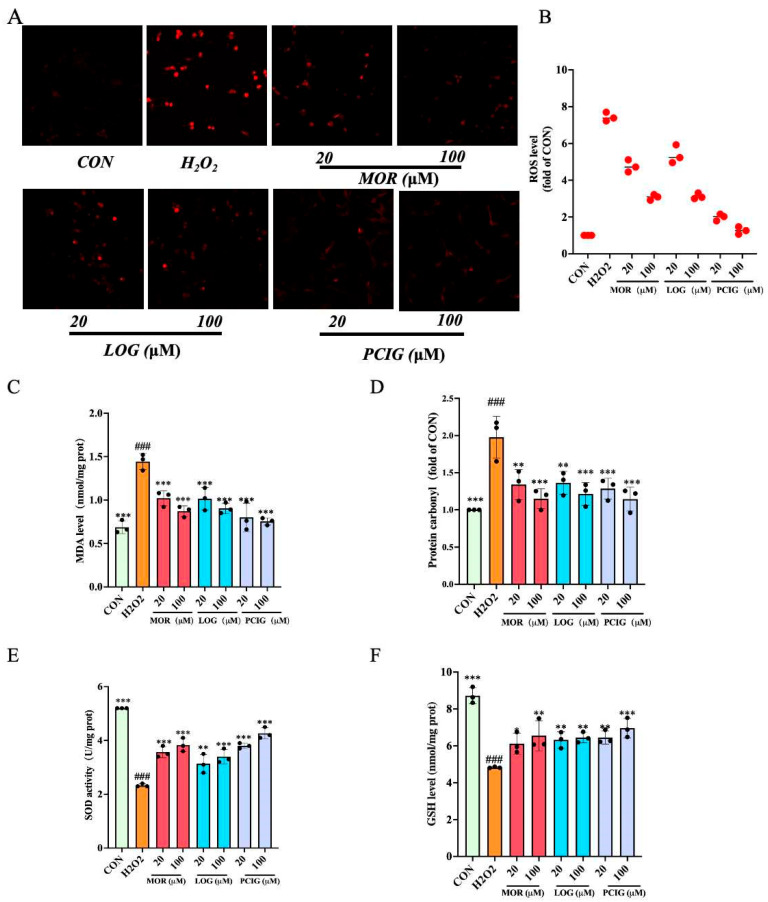
Effects of MOR, LOG and PCIG on reducing oxidative stress induced by H_2_O_2_. (**A**) Representative image of cells with ROS fluorescence. (**B**) Intensity of ROS fluorescence. Levels of (**C**) MDA. (**D**) Protein carbonyl. (**E**) SOD activity. (**F**) GSH. The columns and errors bars were represented as means ± SD (*n* = 3). (* *p* < 0.05 vs. H_2_O_2_, ** *p* < 0.01 vs. H_2_O_2_, *** *p* < 0.001 vs. H_2_O_2_; ### *p* < 0.001 vs. CON).

ROS is a set of oxygen-containing molecules, including hydrogen peroxide, hydroxyl radical (OH^−^), singlet oxygen (^1^O_2_), and superoxide (O_2_^−^). Its accumulation in cells could be detected using a DCFH-DA fluorescent probe. Compared to the untreated PC12 cells (CON), H_2_O_2_-stimulated cells exhibited strong red fluorescence, indicating an increase in ROS production (Figure 7A,B). Treatment with MOR, LOG, and PCIG reduced the number of fluorescent cells in a dose-dependent manner, demonstrating their abilities to inhibit ROS accumulation.

MDA and protein carbonyl are the main toxic by-products produced from ROS accumulation. Compared to untreated PC12 cells (CON), H_2_O_2_ stimulation significantly increased the levels of MDA (Figure 7C) and protein carbonyl (Figure 7D) by 1.10-fold (*p* ≤ 0.0001) and 0.98-fold (*p* ≤ 0.0001), respectively. Treatment with MOR, LOG, or PCIG resulted in a dose-dependent decrease in both MDA and protein carbonyl levels. Compared to the H_2_O_2_-stimulated cells, the MDA level in the cells treated with MOR, LOG, or PCIG at two concentrations showed a decrease of 29.3–39.7%, 29.8–37.4%, and 44.6–47.8%, respectively. The protein carbonyl levels were reduced by 32.2–41.8%, 31.0–38.6%, and 35.0–42.1%, respectively.

In addition, we measured the protein levels of primary enzymes using commercially available kits. For SOD levels (Figure 7E), H_2_O_2_ stimulation resulted in a significant reduction of 55.1% (*p* ≤ 0.0001) in PC12 cells. Treatments with MOR, LOG, and PCIG at two concentrations increased the levels in a dose-dependent manner. Compared with the H_2_O_2_-stimulated cells, the cells treated with MOR, LOG, or PCIG showed increases of 52.8–64.3%, 34.3–45.7%, and 62.8–82.9%, respectively. In the GSH levels (Figure 7F), H_2_O_2_-stimulated cells showed a decrease of 44.7% (*p* ≤ 0.0001). Compared with the H_2_O_2_-stimulated cells, the cells treated with MOR, LOG, or PCIG at two concentrations displayed an increase of 26.7–35.7%, 31.0–33.7%, and 33.7–44.2%, respectively.

### 3.6. Activated Signaling Pathways of Nrf2/NQO-1 and Nrf2/HO-1

**Figure 8:**
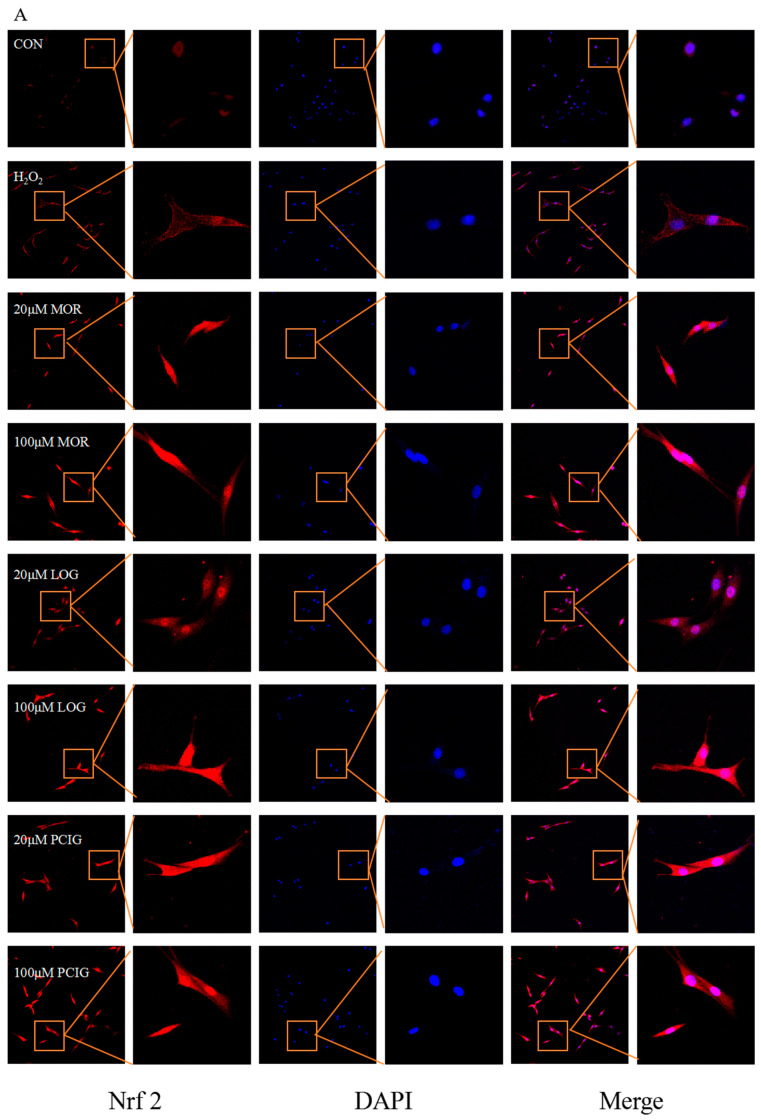

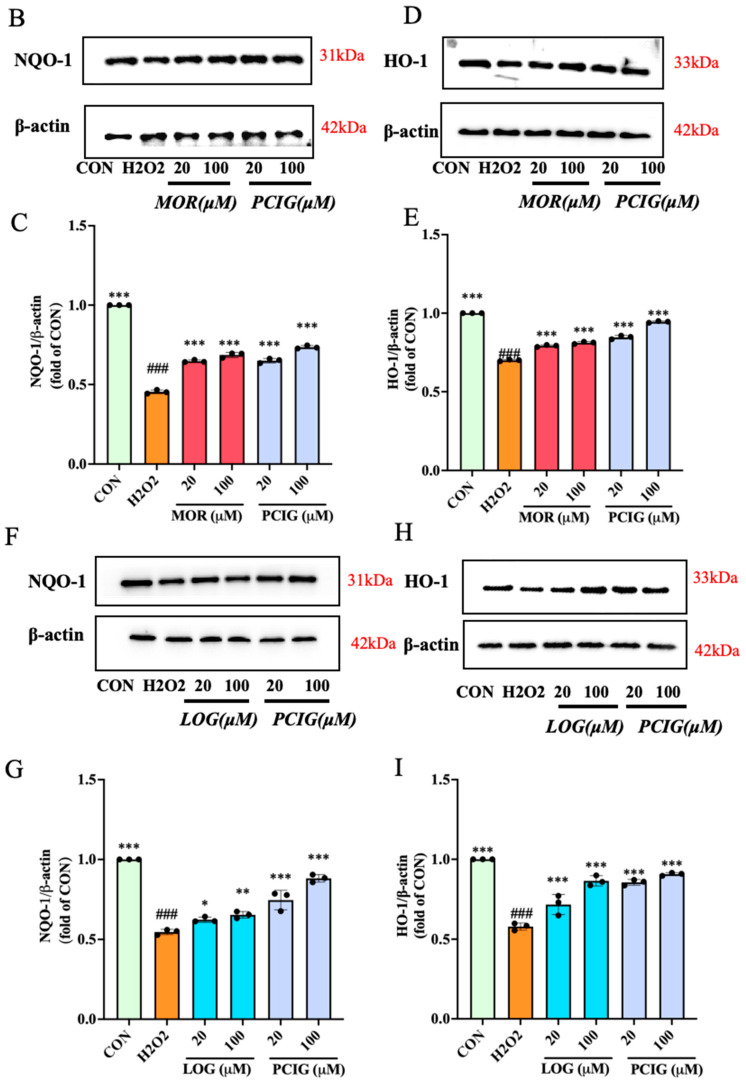
MOR, LOG and PCIG enhanced antioxidative activities in H_2_O_2_-stimulated PC12 cells (**A**) Nrf2 nuclear translocation. The protein levels of (**B**,**D**) NQO-1, (**C**,**E**) HO-1 in H_2_O_2_-stimulated PC12 cells treated with MOR and PCIG. The protein levels of (**F**,**H**) NQO-1, (**G**,**I**) HO-1 in H_2_O_2_-stimulated PC12 cells treated with LOG and PCIG. The columns and errors bars were represented as means ± SD. ### *p* < 0.001 vs. the CON, * *p* < 0.05 vs. the H_2_O_2_, ** *p* < 0.01 vs. the H_2_O_2_, *** *p* < 0.001 vs. the H_2_O_2_ (*n* = 3).

The Nrf2 signaling pathway plays a significant role in helping cells resist oxidative stress and protect brain tissue. Its activation relies on the translocation of Nrf2 from the cytoplasm into the nucleus, where it can initiate the transcription of downstream detoxifying enzymes like HO-1 and NQO-1 [26]. In comparison to the PC12 cells (CON), H_2_O_2_-stimulated cells had an increase in Nrf2 protein production; however, most of the Nrf2 was localized in the cytoplasm (Figure 8A). Treatment with MOR, LOG, or PCIG at two concentrations resulted in a higher expression of Nrf2 protein in the nucleus, indicating successful translocation from the cytoplasm.

Further analysis of the cells showed the expression of HO-1 and NQO-1. H_2_O_2_ stimulation caused a decrease of 45.4% in NQO-1 expression (Figure 8B,F) and 30.0% in HO-1 expression (Figure 8C,G) in PC12 cells. Treatment with MOR, LOG, and PCIG at two different concentrations increased protein expressions in a dose-dependent manner. This concentration effectiveness suggested that their effects are primarily mediated through Nrf2 activation rather than direct ROS neutralization. Specifically, compared with the H_2_O_2_-stimulated cells, treatment with MOR or PCIG led to an up-regulation of NQO-1 expression (Figure 8D,H), with an increase of 49.7–51.2% or 46.8–62.0%, respectively. For HO-1 expression (Figure 8E,I), MOR or PCIG-treated cells showed an increase of 13.2–16.0% or 20.9–56.6%, respectively. LOG treatment resulted in an increase of 13.7–19.1% in NQO-1 expression and 23.8–49.4% in HO-1 expression. In comparison, MOR (100 μM) preferentially upregulated NQO-1 by 1.51-fold compared to H_2_O_2_-stimulated PC12 cells, with a modest induction of HO-1 at 1.16-fold; LOG (100 μM) preferentially activated HO-1, showing a 1.49-fold increase compared to H_2_O_2_-stimulated PC12 cells, but had minimal impact on NQO-1 expression, increasing it by 1.19-fold.

### 3.7. Molecular Docking Studies of Morroniside and Loganin with Keap1

Under homeostatic conditions, Nrf2 is kept inactive by binding to its repressor protein, Keap1 [27]. Disrupting the direct interaction between Keap1 and Nrf2 has been suggested as a mechanism that allows Nrf2 to translocate to the nucleus. The above results indicated that the constituents of PCIG could promote the translocation of Nrf2 to the nucleus. Therefore, molecular docking analysis was carried out to investigate the binding affinity between the compounds and Keap1.

**Figure 9:**
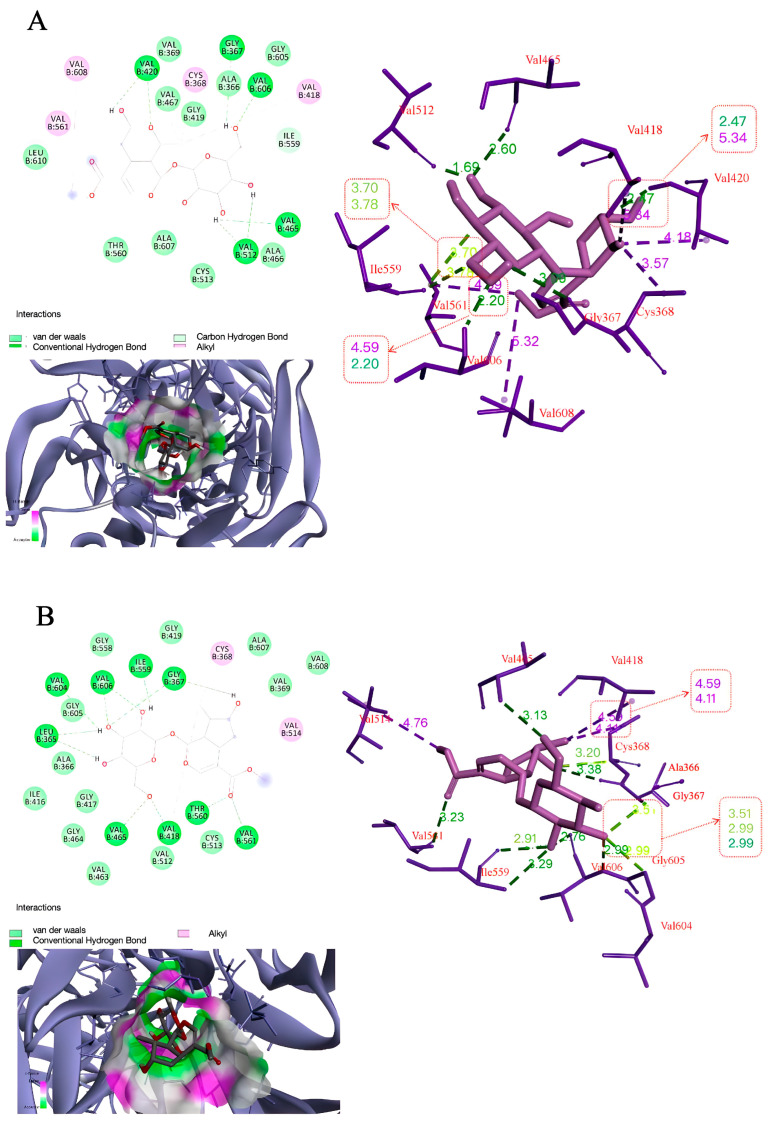
Three-dimensional and partially enlarged graphics of molecular docking analysis for the compounds binding to Keap1 protein (PDB code: 4L7B). The interaction between the Keap1 and (**A**) morroniside; (**B**) loganin.

The results of AutoDock analysis showed that morroniside displayed a binding energy of −5.31 kcal/mol, forming five hydrogen bonding interactions at GLY367, VAL420, VAL465, VAL512, and VAL606 (Table 1, Figure 9A). Loganin exhibited binding energy of −6.54 kcal/mol and interacted with the Keap1 through nine hydrogen bonds at GLY367, ILE559, LEU365, THR560, VAL418, VAL465, VAL561, VAL604, and VAL606 (Table 1, Figure 9B). The results indicated that loganin displayed the lowest binding energy and the greatest number of hydrogen bonding interactions. This suggested that the loganin might have a stronger role in regulating Keap1 than morroniside. Furthermore, the interaction sites in morroniside and loganin were the same, at GLY367, VAL465 and VAL606. This suggested that the two compounds might have a similar fitting region with Keap1.

## 4. Discussion

In stroke research, the MCAO/R injury model is widely used to understand the effects of stroke on the brain. A key aspect is that reperfusion from recanalized cerebral vessels can cause brain edema and the death of brain cells, leading to tissue damage [28,29,30]. The acute response of brain tissue to cerebral ischemia and its chronic progression of pathology involve a complex interplay of various pathways. Growing research highlighted the significant roles of abnormal expression of aquaporin 4 (AQP4), ROS, and inflammation in these processes [31,32,33]. To effectively assess brain injury in animal models, researchers focused on key indicators such as the neurological function, the size of cerebral infarction, and the degree of brain edema. Consistent with previous studies, the MCAO/R rats in this study had severe cerebral damage and cell death occurrence in the brain one hour after blood flow restoration following occlusion [34]. In our earlier study, we demonstrated the beneficial effects of PCIG in reducing brain edema, which was related to the modulation of polarized AQP4 expression [20]. Additionally, evidence suggested that the regulation of *Aqp4* gene expression might occur through various transcription factors activated by oxidative stress [35]. In the current study, the presence of oxidative stress in the MCAO/R rat’s brain was evident through an increase in levels of biologically toxic products (MDA and protein carbonyl), and a decrease in activities of antioxidant enzymes (SOD and GSH-Px). The treatments with PCIG, MOR, and LOG showed neuroprotective effects demonstrated by improved neurological outcomes, reduced infarct size, decreased brain edema, and a lower number of dead cells in the MCAO/R rats. Additionally, the treatments led to decreases in MDA and protein carbonyl levels, along with increases in levels of SOD, GSH, and GSH-Px. These results suggest the potential of PCIG, MOR, and LOG in alleviating the oxidative stress associated with ischemic stroke.

It is widely recognized that drugs can be converted into complex metabolites in the gastrointestinal tract following oral administration. This conversion often results in lower bioavailability, which can influence the drug’s effectiveness. For instance, studies indicated that the oral bioavailability of MOR is approximately 3% [36], while that of LOG is about 5.5% [37]. In contrast, administering drugs intravenously allows them to bypass the digestive process, enabling more efficient delivery to targeted tissues. In this study, we employed oral PCIG and intravenous MOR/LOG concurrently to make a comparison. Three doses of PCIG were selected based on the adult dosage of Corni Fructus described in the Chinese Pharmacopoeia [16], as well as two effective doses identified in our previous study [20]. The dosages for MOR and LOG were determined based on relevant data from the literature [38]. Our results revealed a dose-response relationship with PCIG treatments in MCAO/R rats. Notably, a medium dose of PCIG exhibited greater effectiveness than the individual compounds, MOR and LOG. HPLC analysis confirmed that the medium dose of PCIG corresponded to 30 mg/kg of MOR and 15 mg/kg of LOG, while the intravenous doses of MOR and LOG were 46.2 mg/kg and 18.5 mg/kg, respectively. This indicated that PCIG might offer a more pronounced effect than either MOR or LOG alone. To better understand the effectiveness of the parent PCIG and its components, while minimizing the interference from metabolites generated during oral administration, we conducted a series of cell-based experiments using PC12 cells. Interestingly, PCIG provided protective effects against H_2_O_2_ stimulation that were comparable to those observed with MOR and LOG. This finding suggested that the pharmacological benefits of oral PCIG might arise from the synergistic interaction of MOR, LOG, and other constituents, including their metabolites formed in the gastrointestinal tract.

With the aid of cell protection, mitochondria are known to serve as the energy production centers in cells as well as to play a crucial role in managing oxidative stress [39,40,41]. When oxidative stress occurs, excessive ROS and oxidants can damage mitochondria, triggering intrinsic apoptosis [42]. This cell death process may start with a rapid depolarization of ΔΨ_M_, which in turn causes the release of pro-apoptotic factors from the intermembrane space, setting off a cascade of cell apoptosis signaling events [43]. Central to this apoptotic process are caspases and BCL-2 protein families. The caspase family acts in a cascade manner, with Caspase 3 being a critical factor in executing apoptosis. The BCL-2 family includes both pro-apoptotic proteins and anti-apoptotic proteins that interact to regulate the permeability of the mitochondrial outer membrane. For instance, BAX is a pro-apoptotic protein that could promote cell apoptosis. Its accumulation could trigger the activation of Caspase 3, leading to cell death. Conversely, BCL-2 is an anti-apoptotic protein that could act as a “main switch” to inhibit cell death [44]. This indicated that excessive H_2_O_2_ interfered with the mitochondrial electron transport chain, increasing ROS production and inducing lipid peroxidation, as well as a loss of ΔΨ_M_. This damage prompted the permeabilization of the mitochondrial outer membrane through the activation of pro-apoptotic BCL-2 family proteins and the inhibition of anti-apoptotic proteins. Treatment with MOR, LOG, and PCIG inhibited the intrinsic apoptosis by reducing the production of ROS, MDA, and protein carbonyl. Additionally, these treatments also prevented the loss of ΔΨ_M_ and lowered the BAX/BCL-2 ratio. Our results suggested the potential role of PCIG and its constituents in protecting mitochondria from apoptosis, likely through the suppression of ROS. 

In addition, to combat the accumulation of ROS, cells are equipped to produce various antioxidant defensive enzymes, including primary enzymes (SOD, GSH-Px, and CAT) and inducible detoxifying enzymes (HO-1 and NQO-1) [45]. SOD, GSH-Px, and CAT are essential for combating oxidative stress by eliminating free radicals. HO-1 and NQO-1 induction can render cells more resistant to the potential subsequent challenges of greater stress. HO-1 traditionally refers to the enzymes catalyzing the conjugation of reactions and is closely linked to the function and overall health of mitochondria by participating in the oxidative phosphorylation process within cells. To maintain redox homeostasis in tissues, HO-1 activation is essential in response to various cellular stress conditions such as inflammation, hypoxia, or hyperthermia [46,47]. Boosting the expression of HO-1 protein can effectively reduce the production of harmful hydroxyl radicals by degrading heme, thereby protecting cells from DNA damage and preventing related apoptosis [48]. NQO-1 functions to reduce toxic quinones, helping to eliminate superoxide. Increasing NQO-1 levels could effectively inhibit excessive ROS buildup and safeguard cell membranes from lipid peroxidation [49]. Available data showed that the expression of these enzymes is primarily regulated by the transcription factor Nrf2 [50].

It is well known that Nrf2 exists as part of inactive cytoplasmic complexes bound by Keap1 under homeostatic conditions [51]. Keap1 facilitates the rapid proteasomal degradation of Nrf2 and serves as a sensor for oxidative and electrophilic stress. When the interaction between Nrf2 and Keap1 is disrupted by small molecules, Nrf2 is able to be free and migrates into the nucleus [27]. In the cell-based experiments conducted in this study, treatment with MOR, LOG, or PCIG led to the translocation of Nrf2 from the cytoplasm to the nucleus, and the subsequent activation of downstream pathways such as HO-1 and NQO-1. Molecular docking analysis indicated that MOR and LOG had a strong binding ability to Keap1. This interaction likely served as one mechanism by which Nrf2 could be released from its inactive complex, enabling its movement into the nucleus. Once in the nucleus, Nrf2 could induce the expression of a network of cooperating enzymes, including NQO-1 and HO-1, which are involved in antioxidants and defence proteins [52]. Therefore, the drugs that activate the Nrf2/HO-1 and Nrf2/NQO-1 pathways have been shown to enhance the tolerance of brain tissue to oxidative damage caused by CIR [53,54].

Recent research has highlighted the promising beneficial effects of both non-enzymatic and enzymatic antioxidants in mitigating the developmental programming of ischemic stroke. Non-enzymatic antioxidants could act as scavengers by donating electrons to stabilize free radicals, thus preventing damage to other molecules. However, there are disadvantages to using non-enzymatic antioxidants in stroke treatment. For instance, the increased oxidative stress during stroke can deplete these antioxidants faster than they can be replenished, leading to a deficiency. In some cases, non-enzymatic antioxidants might even behave as pro-oxidants, potentially worsening oxidative damage [55]. Enzymatic antioxidants, such as CAT and GSH, are essential for neutralizing ROS. These enzymes facilitate specific reactions that convert harmful ROS into less damaging molecules. An example is N-acetylcysteine (NAC), a precursor to cysteine, which helps scavenge free radicals by replenishing glutathione [56]. Mitoquinone mesylate (MitoQ) is another noteworthy antioxidant, a mitochondria-targeted antioxidant that specifically reduces mitochondrial oxidative stress [57]. These traditional antioxidants have been investigated for their potential in stroke treatment. However, as noted in analyses of clinical trials, the antioxidants often fail to improve outcomes, possibly due to non-selective ROS scavenging, poor bioavailability, or a temporal mismatch with peaks of oxidative stress [58,59]. Consequently, a multi-antioxidant approach has been suggested as a more effective strategy for managing oxidative stress in stroke [60]. In this study, treatments with MOR, LOG, and PCIG significantly increased levels of SOD and GSH in a dose-dependent manner, and promoted the translocation of Nrf2 into the nucleus. Notably, unlike conventional antioxidants, PCIG could enhance cellular antioxidant capacity globally by activating the Keap1-Nrf2 pathway, making it a potential Nrf2 activator with longer-lasting effects and redox-balanced effects. Additionally, MOR and LOG selectively enhanced the expression of NQO-1 and HO-1, respectively. This selectivity in their pathways may indicate a functional redundancy in Nrf2 activation. Therefore, the pharmacological advantage of PCIG likely arises from its ability to modulate both pathways simultaneously, as using individual compounds might be insufficient to fully address diverse oxidative insults. These findings implied that PCIG, rather than any individual components, might represent a valuable treatment option for ischemic stroke. Further research is necessary to gain a comprehensive understanding of the interaction between the components and targets involved in these processes.

Overall, this study has effectively demonstrated the neuroprotective effects of PCIG against CIR/I, primarily by reducing oxidative stress. The findings from both intravenous administration and in vitro cell-based assays indicated that the key bioactive components, MOR and LOG, exerted their effects by modulating the Nrf2/HO-1/NQO-1 signaling pathway. However, the study still has limitations. For example, it would be beneficial to investigate the effectiveness of combining MOR and LOG in specific proportions. Additionally, the interaction between MOR/LOG and Keap1 or AQP4, as shown in the molecular docking analysis of our current and previous studies, needs further investigation through various experiments, such as competitive binding assays. Moreover, the in silico binding does not guarantee functional disruption of the Keap1-Nrf2 complex in vivo. Further structural studies are needed to confirm whether MOR/LOG binding directly displaces Nrf2 from Keap1 or induces conformational changes.

## 5. Conclusions

**Figure 10:**
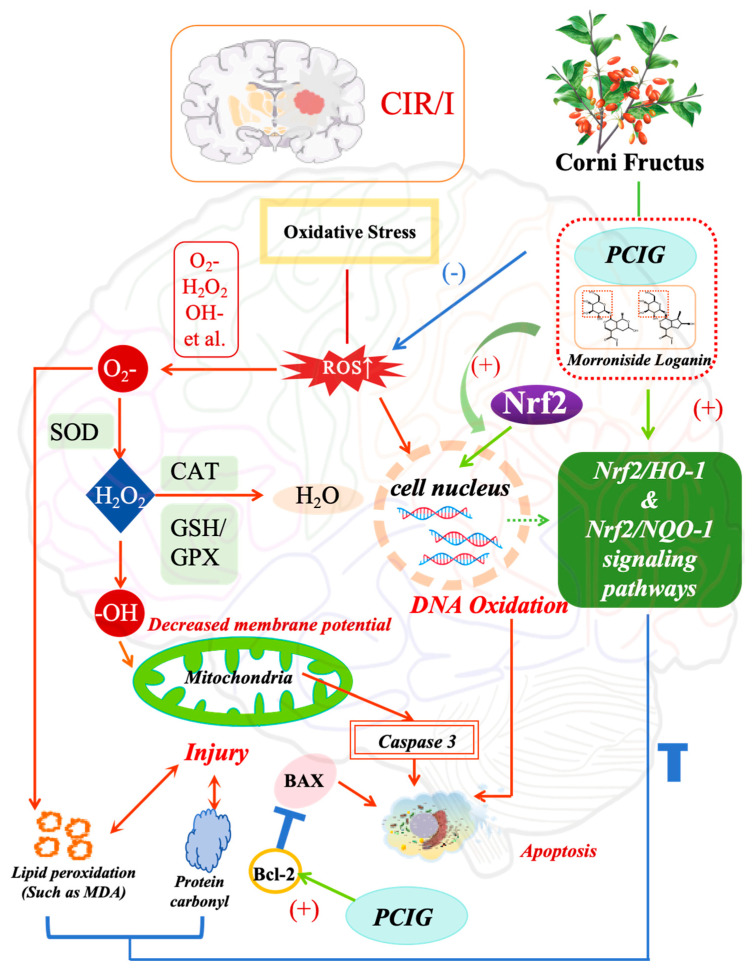
Schematic presentation of the treatment pathway of cerebral ischemia-reperfusion injury with purified cornel iridoid glycosides.

In summary, the oral administration of PCIG effectively protected the rat brain against CIR/I by modulating the Nrf2 signaling pathways. This protective effect was achieved through several mechanisms that reduced oxidative damage and apoptosis: (1) decreasing the production of ROS, MDA, and protein carbonyl; (2) improving mitochondrial function; (3) enhancing the activity of endogenous antioxidant enzymes (GSH-Px, SOD); (4) promoting the translocation of Nrf2 into the nucleus (5) modulating the oxidative signaling cascades associated with Nrf2/HO-1 and Nrf2/NQO-1 pathways via LOG and MOR. (Figure 10).

## Figures and Tables

**Table 1:** Molecular docking studies of morroniside and loganin with Keap1.

Binding Energy (kcal/mol)		Hydrogen Bonding Interactions	Distance (A°)
Morroniside	−5.31	GLY367	3.09
		VAL420	2.47, 2.20
		VAL465	2.60
		VAL512	2.22, 1.69
		VAL606	2.20
Loganin	−6.54	GLY367	2.42, 2.56
		ILE559	1.96
		LEU365	2.83, 2.82
		THR560	2.72
		VAL418	2.65
		VAL465	2.23
		VAL561	2.75
		VAL604	2.96
		VAL606	2.06, 2.44

## Data Availability

Data are contained in the article.

## Abbreviations

ARE, antioxidant response element; CAT, catalase; CIR/I, cerebral ischemia-reperfusion injury; CON, control group; DMEM, Dulbecco’s modified eagle medium; DMSO, dimethyl sulfoxide; GSH, glutathione; GSH-Px, glutathione peroxidase; H&E staining, Hematoxylin and eosin staining; HO-1, heme oxygenase 1; LOG, loganin; MCAO/Reperfusion, middle cerebral artery occlusion/reperfusion; MDA, malondialdehyde; MOR, morroniside; NQO-1, NAD(P)H quinone dehydrogenase; Nrf2, Nuclear factor erythroid-2 related factor 2; PCIG, purified cornel iridoid glycoside; ROS, reactive oxygen species; SD rat, Sprague Dawley rat; SOD, superoxide dismutase; TTC, 2,3,5-triphenyltetrazolium hydrochloride; TUNEL, Terminal deoxynucleotidyl transferase dUTP nick end labeling; AQP4, aquaporin 4.
